# Suicide in older adults in Honduras: a retrospective analysis (2008-2022)

**DOI:** 10.3389/fpsyt.2024.1489874

**Published:** 2024-11-26

**Authors:** María José Irías Escher, Virna Julisa López Castro, Pablo Yup de León

**Affiliations:** ^1^ Escuela de Ciencias Psicológicas, Facultad de Ciencias Sociales, Universidad Nacional Autónoma de Honduras, Tegucigalpa, Honduras; ^2^ Grupo de Investigación en Neurociencias Aplicadas, Universidad Nacional Autónoma de Honduras, Tegucigalpa, Honduras; ^3^ OWSD Honduran Chapter, Organización de Mujeres en la Ciencia para el Mundo en Desarrollo (OWSD), Tegucigalpa, Honduras; ^4^ Escuela de Biología, Facultad de Ciencias, Universidad Nacional Autónoma de Honduras, Tegucigalpa, Honduras; ^5^ Instituto Universitario de Democracia, Paz y Seguridad, Facultad de Ciencias Sociales, Universidad Nacional Autónoma de Honduras, Tegucigalpa, Honduras

**Keywords:** mental health, epidemiology of suicide, depression, suicide in elderly, older adults, suicide methods

## Abstract

**Introduction:**

This study examines suicide among older adults in Honduras over a 15-year period (2008-2022).

**Methods:**

Data were collected from the National Violence Observatory attached to the University Institute of Democracy, Peace and Security of the National Autonomous University of Honduras (ONV-IUDPAS-UNAH), and 593 suicide cases were analyzed with a quantitative approach of descriptive scope, to identify suicide decedent characteristics and patterns in the cases.

**Results:**

It was observed that 94.1% of the suicide decedents were male, with an average age of 70 years, predominantly from urban areas; the highest prevalence was in 2021 (7.77), generally in the mornings and mostly by hanging or asphyxiation in private spaces.

**Discussion:**

There is a significant difference in the prevalence of suicide by gender (16 males per female). Similar to worldwide reports, an increase in post-pandemic suicides is observed. The aging of the population and the increasing incidence of suicide in older adults gives relevance to this study, which has been limited by the lack of systematic data collection and previous research that would allow a better understanding of the problem and, in turn, the generation of public policies focused on the mental health of older adults.

## 1 Introduction

The World Health Organization (WHO) estimates that around 703,000 people die by suicide each year worldwide, 77% of which occur in low- and middle-income countries (1) such as Honduras. Between 2015 and 2019, more than 93,000 suicides were reported in the Americas (2), of which 1,801 occurred in Honduras, the country with the lowest suicide rate in Central America (3).

Globally, the age-standardized suicide rate is estimated to be 2.3 times higher in males than in females (1). While in the Americas, 79% of suicide decedents are males (2), in Honduras, this figure rises to 83% (4). In terms of age, the highest suicide rate in the American region is observed in people aged 45 to 59 years, followed by the population aged 70 years and older (2). In Honduras, according to ONV-IUDPAS by 2021 the highest incidence of suicides corresponds to persons aged 30 years or below, while only 62 suicide decedents (59M:3F) over 60 years were recorded.

Population aging is a growing global concern, especially regarding suicide among older adults. By 2050, the global proportion of people aged 60 or older is expected to nearly double from 12% to 22% (5). Honduras is a Low-Middle Income Country (LMIC) with a population of 10.593.798 people (6). According to data from the University Demographic Observatory (ODU-UNAH), the aging rate ranges from 20 to 32 elderly for every 100 children and is projected to reach 79 by 2045 (7). Locally, these figures are influenced by socio-economic inequalities, high rates of poverty and unemployment, violence, social exclusion, forced migration, and the stigma associated with corruption and crime. These factors, coupled with the predominantly Christian religious landscape, contribute to the unique challenges faced by older adults in Honduras.

There are few data and few studies on suicide among older adults in the Honduran population. In 1992, a study based on the general suicide registry found that 4% of the suicide decedents were adults aged 60 years and above, the lowest percentage of the analyzed group (8). In another study, Palacios (9) characterized the suicide cases registered between 2015 and 2017 at the Institute of Forensic Sciences in Tegucigalpa and identified that 6.2% of the suicide decedents were people aged 63 to 82 years. In both studies, variables such as sex, geographic location, and suicide methods were addressed, but the data are not specific to the older adult population.

Studies worldwide have identified risk factors and epidemiological characteristics of suicide in older adults; depressive disorders predominate among them, followed by other mental disorders such as anxiety, bipolar disorder, dementia and schizophrenia. Loneliness, physical illness, substance use and abuse, economic problems, marital and family problems, among others, are also included. Of all of them, Conwell et al. (10) and Obuobi-Donkor et al. (11) highlight depression as a key risk factor (greater than physical illness and family problems). On the other hand, male sex, violent self-injury, psychiatric disorders, poor medical conditions, stressors, chronic somatic diseases and living alone predict deaths by suicide (12–15).

Depression is more prevalent in females; however, the suicide fatality rate increases with age in males, but not in females (16). This is associated with the fact that males tend to use more lethal methods and are more reluctant to seek psychological help (11, 17). According to WHO (1) the most common method worldwide is pesticide poisoning and the use of firearms. Other studies add hanging to these methods. (11, 16, 18).

Although all these data provide useful information for the understanding of suicide in older adults, there are no studies available in Honduras. The objective of this study is to describe the occurrence of suicide in people over 60 years based on the records of the National Violence Observatory attached to the University Institute of Democracy, Peace and Security of the National Autonomous University of Honduras (ONV-IUDPAS-UNAH) from 2008 to 2022. Beyond the limitations related to the limited data available, it is expected that this study will serve as a basis for further research, which will favor the evaluation, design and implementation of prevention plans in the future.

## 2 Methods

The data were collected from the ONV - IUDPAS - UNAH which takes part of an inter-institutional table of violent deaths made up of the National Police (PN), the General Directorate of Forensic Medicine (DGMF) of the Public Prosecutor’s Office (MP) and the National Registry of Persons (RNP). This table records deaths in a database and validates the variables of time, person and place, case by case, of homicides, suicides, traffic events and unintentional events. The age and sex of the suicide decedents as well as the year, month, day and time of the event are recorded consistently and in a non-systematic way the marital status, area (rural/urban), situational context (conflicts/mental illness), the mechanism of death, place of the event (public/private).

The DGMF is who provides the cause and the mechanism of death according to the autopsies performed; on the other hand, the PN is who provides the contexts of suicide, from informants at the scene, such as relatives, neighbors, acquaintances, among others. Therefore, this section usually has gaps. In cases where the autopsy information is not decisive to identify the type of death, for example, suicide, the ONV - IUDPAS - UNAH classifies it as “unknown intent” and defines it as: “those where official sources have not determined the manner of death because some link in the criminal investigation chain is pending.” (19). The ONV - IUDPAS - UNAH sends a non-compliance report to official institutions with the list of cases of deaths of unknown intent for investigation. Until an official response is obtained, the ONV records remain the same. In this study only the cases where the official source classifies the death as suicide are presented.

This study did not require an ethic approval since the data are public or available under request at ONV-IUDPAS-UNAH guaranteeing the confidentiality and anonymity of the suicide decedents ^1^.

**Figure 1:**
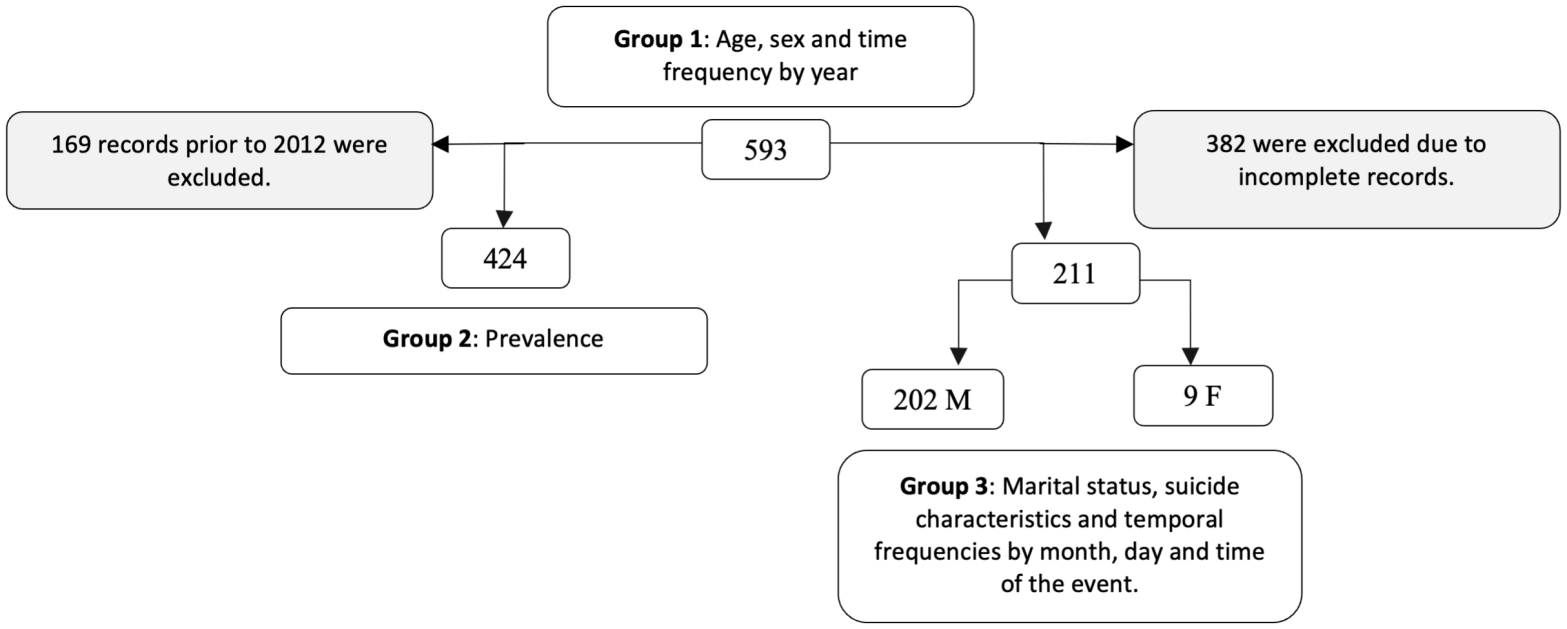
Structure of the data by groups.

The available data were structured in three groups (**Figure 1**). The first group includes 593 cases corresponding to the total registry of individuals who died by suicide over 60 years old registered in the period 2008 - 2022; with this group, the variables of age was divided in third age: defined as a person who has reached sixty (60) years of age and fourth age: defined as a person who has reached eighty (80) years of age or older (20, 21), for a detailed analysis, the third age were divided into five-year periods (**Table 1**), sex of the suicide decedent and temporal frequency by year were analyzed. The second group consists of 424 cases resulting from the exclusion of 169 records prior to 2013, since there are no adjusted population projections according to the 2013 national census; with this group the prevalence analysis was performed. The third group contains 211 (202M:9F) cases resulting from the exclusion of 382 incomplete records in at least one of the following variables: marital status, characteristics of suicide (zone, context, location and mechanism), and temporal frequencies by month, day and time of the event; these were the variables analyzed in this group.

**Table 1:** Suicide decedent characteristics by sex and age, Honduras, 2008-2022.

	Sex					
	Males		Females		TOTAL	
	n	%	n	%	n	%
Age in years (n=593)						
**Third age**						
60-64	160	28.7%	13	37.1%	173	29.2%
65-69	131	23.5%	6	17.1%	137	23.1%
70-74	96	17.2%	7	20.0%	103	17.4%
75-79	83	14.9%	7	20.0%	90	15.1%
**Fourth age**						
80 +	88	15.8%	2	5.7%	90	15.1%
Total	558	94.1%	35	6.3%	593	100%
**Marital Status (n = 211)**						
Married, free union	117	57.9%	2	22.2%	119	56.4%
Single, divorced, widowed	85	42.1%	7	77.8%	92	43.6%
Total	202	95.7%	9	4.26%	211	100%

A quantitative approach was used, consisting of a descriptive analysis of cases of suicide in older adults. The data were systematically analyzed using the Jamovi system version 2.3.12 (data processing) and Microsoft 365 Excel (graphs and tables). Groupings and categorizations were performed to identify patterns and statistics were applied to obtain descriptive measures of the variables analyzed. Chi-square tests were also performed to obtain descriptive measures of the variables analysed. Prevalence data were calculated by dividing the number of suicide cases by 100,000 inhabitants aged 60 years and older. For the trend of the data, a linear function was used with the R statistical analysis software. Where the equation of the line is y = -3609.06 + 1.81 * x with an R^2^ of 0.44.

## 3 Results

### 3.1 suicide decedent characteristics

#### 3.1.1 Sex of suicide decedents

Of the 593 cases, the highest incidence (defined as the number of new cases reported each year) occurred in males, with a ratio of approximately 16 male suicides for every one female suicide (**Table 1**) with a chi-square statistical significance of 61.6598. Significant at p <.05.

#### 3.1.2 Age of suicide decedents

The average age of males was 70 ( ± 8) years, with a range between 60 and 96; in females the average age was 69 ( ± 7) years, with a range between 60 and 87. The five-year period with the highest incidence was from 60 to 64 years old with 29.2%, a similar proportion in males (28.7%) but higher in females (37.1%). In general, there are more individuals who died by suicide in the third age group (60 – 79 years old) than in the fourth age group (over 80 years old) in a ratio of 6:1. The incidence rate ratio (IRR) of suicide is 5.25 times higher for the first than for the second in males and 15.66 for females (**Table 1**).

#### 3.1.3 Marital status of suicide decedents

In the 211 cases analyzed (202M:9F), 57.9% of the males were married or in common-law unions, while 77.8% of the females were single, divorced or widowed (**Table 1**).

### 3.2 Prevalence

Of the 424 cases recorded between 2013 and 2022, the highest prevalence for both the third (7.10) and fourth age (11.42) is 7.77, recorded in 2021. In contrast, the lowest overall prevalence is 4.62 in 2013, with a variation between age groups of 4.69 in 2013 for the third age and 2.85 in 2017 for the fourth age (**Table 2**).

**Table 2:** Suicide prevalence rate in older adults, Honduras, 2013-2022 (n = 424).

Year	Population 60 +	Population 3rd age (60-79)	Population 4th age (80 +)	Urban population	Rural population	Victims 60 +	Victims 3rd age (60 -79)	Victims 4th age (80 +)	Victims 60 + urban area	Victims 60 + rural area	Prevalence	Prevalence 3rd age	Prevalence 4th age	IRR	Urban prevalence	Rural prevalence
2013	605,667	511,821	93,846	317,821	287,846	28	24	4	21	5	4.62	4.69	4.26	1.10	6.61	1.74
2014	626,546	529,772	96,774	332,188	294,367	36	33	3	15	21	5.75	6.23	3.10	2.01	4.52	7.13
2015	648,322	548,852	99,470	347,011	301,320	30	27	3	19	11	4.63	4.92	3.02	1.63	5.48	3.65
2016	670,961	568,765	102,196	362,299	308,671	39	33	6	27	12	5.81	5.80	5.87	0.99	7.45	3.89
2017	694,510	589,277	105,233	378,109	316,412	38	35	3	24	14	5.47	5.94	2.85	2.08	6.35	4.42
2018	718,969	610,209	108,760	394,441	324,540	37	31	6	22	15	5.15	5.08	5.52	0.92	5.58	4.62
2019	744,438	631,527	112,911	411,360	333,091	42	34	8	23	19	5.64	5.38	7.09	0.76	5.59	5.70
2020	770,905	653,308	117,597	428,841	342,077	51	45	6	34	15	6.62	6.89	5.10	1.35	7.93	4.38
2021	798,410	675,776	122,634	446,919	351,505	62	48	14	24	15	7.77	7.10	11.42	0.62	5.37	4.27
2022	827,056	699,257	127,799	465,602	361,468	61	52	9	28	20	7.38	7.44	7.04	1.06	6.01	5.53

Prevalence calculated as rate per 100 thousand population.

### 3.3 Characteristics of suicide

After discarding the cases with incomplete information, we proceeded to the analysis of the remaining 211 cases that had complete records available (**Table 3**). According to the area of occurrence, in both sexes, a higher frequency of suicides was identified in the urban area (62.4% M: 77.8% F). When it comes to the context, meaning the potential key contributors to the suicide decedent making the decision to end his or her life, depressive problems are observed as the main motive (71.3%M:100%F). Other contexts include family conflicts and conflicts with a partner.

**Table 3:** Suicide characteristics by sex, Honduras, 2008-2022 (n=211).

		Sex			
		Male		Female	
		n	%	n	%
Zone	Urban	126	62.4%	7	77.8%
	Rural	76	37.6%	2	22.2%
Context	Depressive problems	144	71.3%	9	100.0%
	Family conflict	27	13.4%	0	0.0%
	Conflicts with the partner	21	10.4%	0	0.0%
	Others	10	5.0%	0	0.0%
Location	Private	146	72.3%	8	88.9%
	Public	56	27.7%	1	11.1%
Mechanism	Hanging (asphyxiation)	108	53.5%	4	44.4%
	Firearm	48	23.8%	3	33.3%
	Poisoning	43	21.3%	2	22.2%
	Others	3	1.5%	0	0.0%

Regarding the place of the incident, private spaces were the most common (72.3% M:88.9% F) versus public spaces. The mechanism used was as follows: hanging or asphyxiation (53.5% M; 44.4% F), firearm (23.8% M; 33.3% F) and poisoning (21.3% M; 22.2% F).

### 3.4 Time frequencies

**Figure 2:**
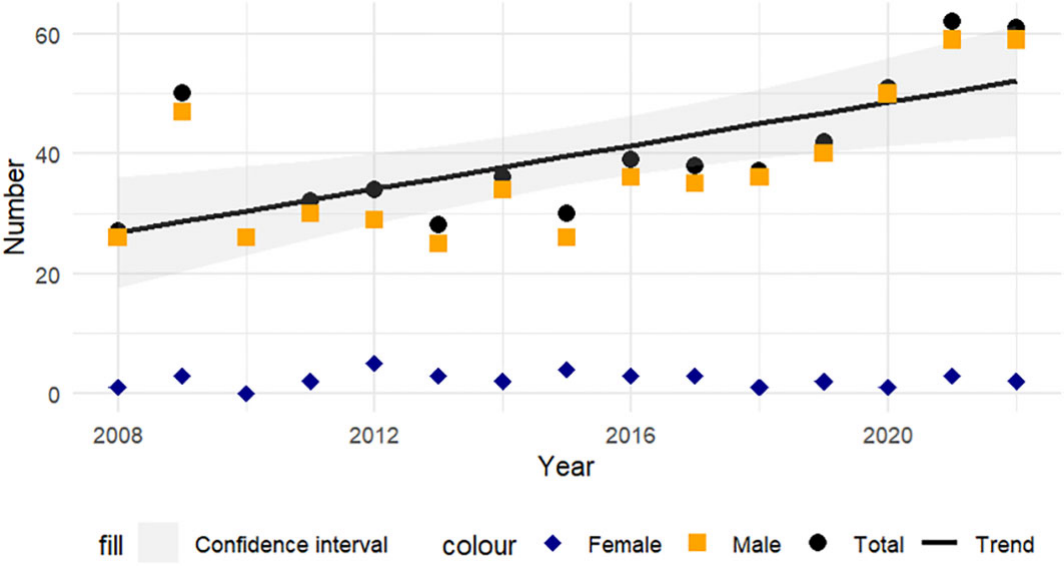
Spatial distribution of suicides by sex, Honduras, 2008-2022.

Taking the 593 cases available, suicide among males and females shows oscillations. Although there is a general increase, there are years with significant fluctuations. For example, after an increase in 2009, there was a drop in 2010. However, from 2014 onward, the trend appears more stable and generally upward. By the year 2024, approximately 56 suicides of older adults would be expected to occur, however, this result is only indicative, given that the goodness of fit of the model is 0.44 (**Figure 2**).

**Figure 3:**
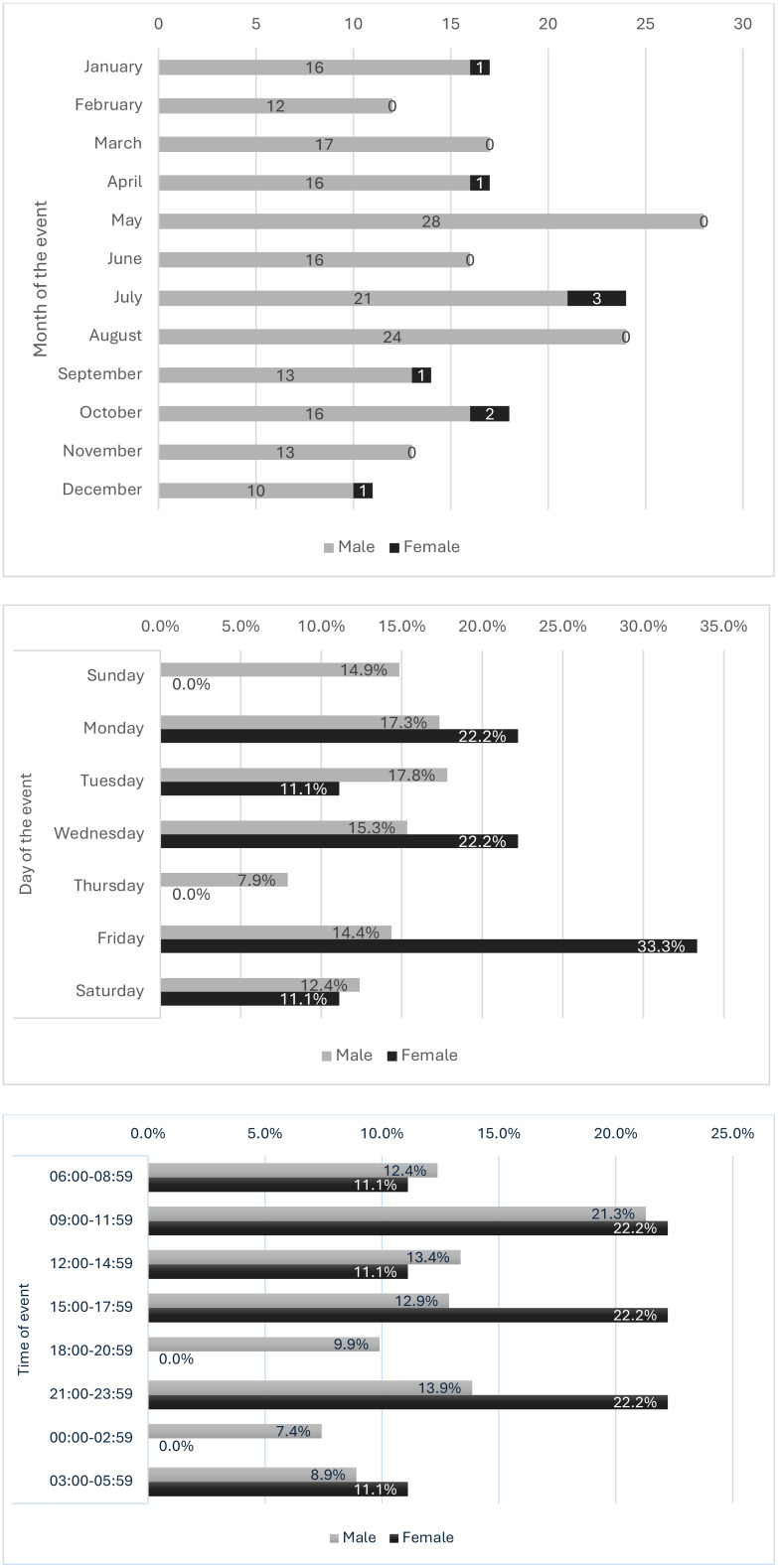
Frequency of suicides by sex according to month, day and time Honduras, 2008-2022 (n = 211).

When analyzing the frequency throughout the year, of the 211 cases analyzed, it is observed that more than 10 males die by suicide every month, but in May this number is 2.8 times higher, followed by August and July (> 20 accumulated cases per month); in females, July and October were the months with the highest frequency. The highest percentage of suicides in males was on Tuesday (18%) and Monday (17%); in females, on the other hand, the highest percentage occurred on Friday (33%). Most suicides (59.9%) occur between 09:00 and 17:59 in both sexes. In males, the time with the highest percentage (21%) is from 09:00 to 11:59; in females, there are three intervals with the same percentage (22%), the first from 09:00 to 11:59, the second from 15:00 to 17:59 and the third from 21:00 to 23:59 (**Figure 3**).

## 4 Discussion

### 4.1 Suicide decedent characteristics

This study examined data on suicides in people aged 60 and above in Honduras between 2008 and 2022, stratified by age and sex. These adults, still functional, face significant evolutionary challenges, such as job retirement and changes in family roles, which may affect their adaptability (22). It was found that 29.2% of cases correspond to people between 60 and 64 years old, and the incidence decreases with increasing age. Although studies such as those of Lee et al. (23) and Razai et al. (24) report similar results, others, such as Garnett et al. (25) and the PAHO/WHO (26) show the opposite in the United States and the Americas region.

The gender disparity in suicide rates is notable, with a significantly higher prevalence in males (94.1%). This finding is consistent with other studies (11, 14, 18, 25, 27) and with data on the general population in Honduras (8, 9). Following the approach of Wen et al. (28) on the Chinese population, further research is suggested to understand this gap in Honduras. Preliminarily, this disparity could be attributed to the fact that 71.9% of males in Honduras are heads of household (29) and retirement from the labor market could entail the loss of traditionally male cultural and social roles, with fatal consequences. These changes are more abrupt for males than for females, who mostly retain roles linked to the home and family regardless of their age.

Most of the males who die by suicide were married or in common-law unions, which contrasts with the overall data for Honduras, where 64% of suicides between 2015 and 2017 corresponded to single people (9). According to data from the Permanent Household Survey of the National Institute of Statistics (30) by 2016, 40.6% of people aged 35 to 59 were married compared to 36.8% of people over 60 years old. In other cases, previous studies, such as those by Goretti et al. (15) and Sadek et al. (31) indicated a higher prevalence of suicide in males who live alone or have experienced a recent loss, such as the death of a spouse. This difference could be linked to the pressures associated with the role of head of household, which persists despite changes in the environment and can trigger family and couple conflicts, leading to depressive symptoms and, ultimately, the decision to die by suicide.

### 4.2 Prevalence

Globally, the suicide rate in older adults ranges from 18-22 per 100,000 males and 3.5-4.5 per 100,000 females (31). In Europe, de Souza Minayo and Cavalcante (32) reported a prevalence of 29.3 per 100,000 people over 65 years old. In contrast, in Honduras, the prevalence in 2022 was 7.38, which is 14.1 times lower than the global rate. This interpretation may be limited by the lack of data and underreporting, recognized problems in Honduras, as well as by stigmatization associated with sociocultural factors (33).

### 4.3 Characteristics of suicide

Wen et al. (28) found that the suicide rate is higher in rural than in urban areas in China. Their analysis highlights that urbanity represents a better financial situation, access to health systems and insurance coverage, while rurality brings loneliness and lack of care for the elderly, as young people migrate to cities. However, in Honduras there was a predominance of suicides in urban areas in both sexes. Unlike China, urbanity in Honduras faces aggravating factors such as violence, insecurity, lack of public recreation areas and inefficient public transportation, which imposes greater challenges for older adults in cities.

Biological factors (such as physical illness, sexual dysfunction, and severe pain) and social factors (such as isolation, economic pressures, infidelity, and living alone) have been identified as leading to depression and, consequently, suicide in older adults. According to Sadek et al. (31) Major Depressive Disorder (MDD) is a key indicator of suicidal behavior in this population. Obuobi-Donkor et al. (11) also highlight depression, mental health disturbances and perceived stress as predictors of suicidal ideation and behavior. In Honduras, the reason for suicide is unknown in most of the cases analyzed in this study (382 of 593), due to the lack of records and previous studies exploring these risk factors. However, available data indicate that most cases are associated with depressive, family or couple problems, which is consistent with other studies (15, 32, 34–39).

Most suicides occurred in private spaces, with 88.9% of cases in females and 72.3% in males. This finding suggests that older adults may choose places where they feel more comfortable and less exposed to social judgment, which is consistent with other studies indicating that suicides in private spaces are common due to the search for privacy and avoidance of associated stigma.

Hanging is, internationally, the most frequent mechanism for suicide (11, 18, 27, 40). Other methods include the use of firearms, poisoning, jumping from heights, electrocution, and traffic accidents. In the Honduran context, it is observed that older adults present a prevalence of hanging, firearms and poisoning in that order, while the general population follows the pattern of hanging, poisoning and firearms (4). The predominance of hanging may be explained by the accessibility of the necessary materials and the high lethality of the method, as suggested by Zeybek et al. (18).

### 4.4 Time frequencies

Analysis of the seasonality of suicides reveals an increasing trend in males, while in females the pattern is less clear. A peak in suicides was observed in older male adults in 2009, the year in which a political situation occurred with the withdrawal of power of the then president of the country, which generated negative social and economic effects that could have affected the mental health of older adults, making them more vulnerable to suicide, however, this study does not have data to confirm this association. The increase observed in 2020 and 2021 is aligned with reports linking this increase to the COVID-19 pandemic, which affected mental health globally and exacerbated psychosocial risk factors such as isolation, economic stress, and anxiety, which can lead to depression and suicidal behavior (28, 31, 41).

Regarding the day of the week and time of the day, it was observed that suicides occur mainly during the week (Tuesday and Monday for males and Friday, Monday and Wednesday for females). These findings differ with the data for the general population, where Sunday is the day of highest incidence (4). A possible explanation for this finding is that older adults may choose weekdays because they are the time when they are alone while their family members work or get educated, which allows them to have more access to materials with which they can die by suicide without being discovered. In addition, most suicides occur in daytime, with 59.9% in older adults and 35.8% in the general population (4).

## 5 Limitations

This study is the first in Honduras to describe the sociodemographic variables of suicide deaths in older adults. It suggests that certain factors contributing to suicide risk may have cultural and socioeconomic aspects, making them relative/contextual factors rather than absolute, hence the need for caution in applying results from other cultures. Although it is informative and provides a baseline, it has some limitations, including the unspecific data, mainly related to the cause of suicide such as mental illness; the possible biases associated to cultural factors, such as religion and stigmatization which favors subrecording; and the substantial limitation of systematic and accurate collection of these data, which is why 64.5% of the cases in this study were lost. These aspects need to be improved in order to carry out more in-depth research and comprehensive approaches that favor a better understanding of this phenomenon (26). This would allow the analysis of trends and the development of multivariate models to identify protective and predictive risk factors. This is the only way to generate and implement public policies and intervention programs aimed at preserving the mental health of older adults, mainly in urban areas where the incidence is higher.

## 6 Conclusion

This study provides significant insight into suicide in older adults in Honduras, a topic little explored in the national scientific literature. Through a retrospective analysis of ONV-IUDPAS-UNAH data, patterns were identified that highlight the high incidence of suicide in older males, the predominance of hanging as a method, and the concentration of cases in urban areas.

These findings, coupled with population aging, underscore the urgent need to address suicide in this population with specific and well-targeted strategies. Interventions should focus on implementing contextualized suicide awareness and prevention programs, mental health promotion, and well-being of older adults. Furthermore, it is critical to improve the collection and analysis of suicide-related data to enable a more detailed and effective understanding of risk factors in this population.

Finally, the need for a coordinated and well-founded response to address the problem of suicide in older adults in Honduras with practical and public policy approaches is clear. Likewise, the training of health professionals should be strengthened to identify and treat early signs of suicide risk in this population. It is suggested that future research delve deeper into the analysis of risk factors and effective interventions, with the objective of reducing the incidence of suicide and improving the quality of life of older adults in the country.

## Data Availability

The raw data supporting the conclusions of this article will be made available by the authors, without undue reservation.

## Footnotes

1 Available data as fact sheets at https://iudpas.unah.edu.hn/areas/observatorio-de-la-violencia/boletines-del-observatorio-2/boletines-nacionales/ and open raw data online at https://iudpas.unah.edu.hn/areas/observatorio-de-la-violencia/datos-abiertos/.
